# The New York Institute of Stomatology

**Published:** 1898-04

**Authors:** 

**Affiliations:** The New York Institute of Stomatology


### Reports of Society Meetings.

### THE NEW YORK INSTITUTE OF STOMATOLOGY.

A REGULAR meeting of the Institute was held Tuesday evening, February 1, 1898, at the office of Dr. Charles O. Kimball, 27 West Thirty-eight Street, New York City, the President, Dr. E. A. Bogue, in the chair.

The minutes of the previous meeting were read and approved.

*The President.*—Our fellow-member, Dr. George S. Allan, has consented to bring before us this evening a paper upon the use of hydrogen dioxide in its different strengths in the treatment of pulpless teeth.

(For Dr. Allan's paper, see page 201.)

*The President.*—Discussion of Dr. Allan's paper is now in order. Gentlemen present who are not members of the Institute will please consider that they are cordially invited to join in the discussion.

*Dr. S. C. G. Watkins.*—I would like to ask Dr. Allan one or two questions. He showed us how he would dip the piece of cotton into pyrozone, allowing the ether to evaporate, pure $H_2O_2$ remaining on the cotton. I would like to ask him how long that would remain in a root with putrescent contents without becoming offensive or losing its power.

*Dr. Allan.*—That is a question I could not answer; it would depend entirely upon the relative amounts of hydrogen dioxide and putrefying substances in the pulp-chamber. If there were a surplus of the $H_2O_2$, of course there would be no putrefaction whatever.

*Dr. Watkins.*—I would also ask if, in treating deciduous teeth, Dr. Allan would carry the cotton into the canals and allow it to remain there.

*Dr. Allan.*—I would carry the cotton into the canal only while treating a tooth; that is to say, my whole object would be to get the roots of the tooth in an aseptic condition, pure and healthy, and all germs of putrefaction removed. I would not attempt to fill them, but would close the open ends of the roots with gutta-percha, which seems to be about the best material, first using some varnish to keep germs out of the dentinal tubules.

*Dr. Watkins.*—After bleaching teeth with pyrozone, does Dr. Allan protect them with any kind of varnish before filling?

*Dr. Allan.*—Yes; I use cavatine of the S. S. White Company. I do not know what it is made of, but it seems to me to be effective. Anything of that kind is useful to a great degree in keeping germs from entering the tubules.

*Dr. L. L. Howell.*—If Dr. Allan opened into a tooth containing a putrescent pulp, would he apply the hydrogen dioxide in full strength before he removed the pulp?

*Dr. Allan.*—I think under those circumstances it would be a very good plan to employ the three-per-cent. solution cautiously, to do away with the offensive odor and the annoyance of handling a pulp in that condition. The sulphuric acid treatment, introduced by Dr. Callahan, affords a very valuable method of treating putrescent pulps. I know of nothing that gives better results than the sulphuric acid and soda treatment, but in the ordinary operations of treating dead teeth my plan is to open them as carefully as possible, syringe them out with warm water first and then with pyrozone, not attempting to put in cotton or in any way induce pressure towards the end of the root. After washing the root out as well as possible, I put in a very loose pledget of cotton with borolyptol, oil of wintergreen, or some similar preparation upon it. After a while the fluid contents of the pulp-chamber and roots should be drawn off with small rolls of absorbent paper, and a loose pledget of cotton soaked in some strong germicide should then be sealed up in the cavity. I think Dr. Howe made some remarks about this method of treating pulpless teeth a few years ago We should always be very careful not to force any of the contents of a putrescent root through the foramen, and it takes very little pressure to do it.

*Dr. Watkins.*—When would Dr. Allan use the twenty-five-per-cent. solution of hydrogen dioxide?

*Dr. Allan.*—Not in a living tooth, and not in a dead one until the foramen had been completely closed. If we use the ethereal preparations of $H_2O_2$ in a dead tooth without first sealing the foramen, we will have many reasons to regret our lack of caution. We can use the three-per-cent. aqueous solution of pyrozone with a great deal more freedom, but even with that the best practice is to seal up the foramen.

*Dr. W. St. George Elliott.*—A great many prominent men in the profession have advocated the use of cataphoresis in bleaching teeth, and a good many of us have met with considerable success. I am not at all surprised that Dr. Allan failed. I am rather surprised that any of us have ever met with success, because we have always used the wrong pole of the battery; we have used the positive pole when we should have used the negative, and our success has been owing to the pyrozone and not to cataphoresis. The younger members of the profession have no idea of the position they are in now as compared with those who entered the profession many years ago. Of course we know that antiseptic surgery is a modern idea. We attribute it largely to Mr. Joseph Lister, now Sir Joseph Lister, a personal acquaintance of mine whom I esteem very highly. His introduction of the system, while it was original so far as he was concerned, was very quickly abandoned for more simple means. It happened to be my fortune at one time to be a surgeon in the army; I had charge of the Ninth Army Corps Hospital, with many wounded. We had an enormous amount of gangrene and gangrenous trouble of every kind. We had great difficulty in treating it, and had to resort to pure bromine, nitric acid, etc. In those days it was impossible to open the joints without serious results. There were always adhesions, and a stiff joint resulted. Mr. Lister told me that in his first experience he met with that difficulty, but by the use of the antisiptic spray he got rid of it. "Now," I said, "please tell me what is the strength of the carbolic acid that you use." He answered, "Three per cent." I asked, "Will three per cent. destroy micro-organisms?" "No," he answered. Since that time the advance has been such that we have abandoned the spray and gone into the use of antiseptics, and have now even commenced to abandon antiseptics. I was at an operation in the Johns Hopkins Hospital some time ago, and in talking to Dr. Halstead asked him what was the present status of antiseptics in surgery. He replied, "They are a good thing, but they are by no means a necessity; for two years we have run this hospital without the use of any antiseptics whatever, and with perfect success; but it is a great deal easier to use antiseptics than to take the extra precaution of great cleanliness which is otherwise necessary."

*Dr. J. Morgan Howe.*—I would like to ask Dr. Allan how he washes out the root, with a syringe, or by swabbing the canal, or by some other method?

*Dr. Allan.*—I wash it out by swabbing, taking a very little cotton, so that no pressure is required. With a fine needle wrapped with a few fibres of cotton, pyrozone can be forced well down into most of the larger roots, the eye-teeth or the large molars; but to do effective work it should be carried down on a little broach, always looking out not to have too much cotton.

*The President.*—We are to consider the use of pyrozone uncalled for until after the foramen shall have been sealed?

*Dr. Allan.*—It may be used very cautiously, but only the three-per-cent. aqueous solution.

*The President.*—The discussion being closed, we will now hear from Dr. S. C. G. Watkins, of Montclair.

*Dr. S. C. G. Watkins.*—The Institute's Executive Committee having been kind enough to ask me to tell what I know about xeroform, I gladly comply. This is one of the new preparations, which with many operators takes the place of iodoform. I have a few points here which I will read and then give a little experience.

Xeroform, tribromphenol-bismuth, is a new preparation, to take the place of iodoform. It is a very fine greenish-yellow powder, almost odorless and non-irritating. It is very soothing and healing in its effects, and will reduce pus secretions very rapidly, in fact, more rapidly than almost anything I have used. It will promote new granulations and cicatrizations more quickly than most remedies. It is recommended for stopping hemorrhage; is also said to allay pain in cases of burns; it is lighter than iodoform, and does not require as much to spread over a surface. Another strong point in its favor is that it is non-poisonous and can be administered in large doses internally.

The reports from hospital and general practice demonstrate conclusively that xeroform is not to be ranked with many of the so-called substitutes for iodoform, but it is a substance having all the best qualities of that drug in an enhanced degree without its disadvantages, as also a number of other valuable properties that iodoform does not possess. The following comparison shows this plainly:

| IODOFORM. | XEROFORM. |
|---|---|
| Poisonous. | Absolutely non-poisonous. |
| Marked and persistent odor. | Practically odorless. |
| Causes eczema. | Never causes eczema. |
| | Free from germs. |
| Very feeble action upon bacteria. | Very powerful action upon bacteria. |
| Not sterilizable in its ordinary form. | Sterilizable by simple heating. |

I wrote to the importers, asking them how it compared with iodoform as an antiseptic, and they sent me the following statement and comparison. Dr. W. Schmidt, of the University of Berne, made a series of very thorough comparative experiments to determine the bactericide value of a number of the more recently introduced powdered antiseptics. I will quote from his report the following results of the tests, which were made with anthrax cultures, which, as is well known, are considered the most resistant micro-organisms:

"PER CENT. IN MEDIUM.

| | Per Cent. | First Day. | Second Day. | Third Day. |
|---|---|---|---|---|
| Xeroform | .5. | Diminished growth. | Diminished growth. | Diminished growth. |
| Xeroform | 1. | Diminished growth. | Diminished growth. | Diminished growth. |
| Xeroform | 2. | Death. | Death. | Death. |
| Xeroform | 5. | Death. | Death. | Death. |
| Iodoform | .5. | Growth. | Growth. | Growth. |
| Iodoform | 1. | Growth. | Growth. | Growth. |
| Iodoform | 2. | Growth. | Growth. | Growth. |
| Iodoform | 5. | Growth. | Growth. | Growth." |

Xeroform is insoluble in water, alcohol, vegetable oils, fluid vaseline, and chloroform. It is soluble in two per cent. hydrochloric acid, in the proportion of thirty to one hundred.

I have been using it for several months with most marked success, in cases of pulp-canals, in all conditions, giving one treatment and allowing it to remain indefinitely.

I will state a few cases, so that my hearers can see in what way I have been making use of it. In July, just before I went on my vacation, a gentleman called with a second superior bicuspid, crown nearly gone. The canal was decayed and really in a bad state of putrefaction, and the man was suffering from periostitis. I cleansed the canal, pumped in xeroform with a liquid antiseptic, stopped it up with cotton, sealed the cavity with gutta-percha, and went away on my vacation. At the end of eight weeks the patient returned to have further work done. I removed the cotton, which I found to be clean and the xeroform apparently without any deterioration. He stated that he had had no pain after the one treatment. I will say here that that is my general practice. I rarely treat a canal more than once, but in nearly all cases I cleanse the canal as near to the end of the root as I can, syringe it well with some antiseptic, and then pump the xeroform, in connection with some antiseptic, into the canal and stop it up with cotton and sandarac. I always expect to fill at the second sitting, generally giving about ten days between the sittings.

Another case. A gentleman called about a month ago with an abscess which had formed at the end of the palatine root of the right superior wisdom-tooth. I opened the canal and carried the medicine to the end of the root, and then opened the abscess in the roof of the mouth. I filled the canal with xeroform and oil of cinnamon and a rope of cotton, and sealed the cavity with cotton and sandarac. I then washed out the abscess with a two-per-cent. solution of trikresol, then placed a goodly quantity of the xeroform in the abscess cavity, and carried a rope of cotton into the cavity, and allowed it to remain. He came in the second day after that for further treatment, and on removing the cotton, the xeroform was dry and apparently clean. There was still some soreness, and I opened the abscess cavity a little deeper, getting a drop of pus, then washed it out, and carried the xeroform into it the second time. After two days I repeated the operation, and by that time it had almost healed, so that I did not treat it again. The cavity had nearly filled with new granulations, and in the course of a few days was entirely well.

There was a case of a lady who had a spot of eczema on the face, about the size of a cent, that had been there for a long time and had resisted treatment. I gave her a little vial of borolyptol and one of xeroform and asked her to apply them alternately. The borolyptol simply as an antiseptic wash to wet the surface so that the xeroform powder would adhere. She applied them frequently, and at the end of a couple of weeks the spot had almost disappeared.

I also had a case of eczema on the hand, which had been troubling the patient for about a year; there were two cracks forming a cross, which would bleed slightly at every bend of the hand. One Saturday night I bandaged it, first wetting the parts with borolyptol and then placing the xeroform upon it. I treated it again Sunday morning, and again on Sunday night, and on Monday night it was entirely healed, and there has been no return of it.

I also had a case of a man who had torn his wrist badly, among the tendons, on a rusty wire nail in a salt mackerel barrel. He paid no attention to it, and I did not see it for a week after the accident. At that time it was in bad condition, having a greenish cast and was sloughing. I washed it carefully, using a two-per-cent. solution of trikresol, and then applied the xeroform, dressing it once a day. In the course of two or three days a marked change was noticeable, new granulations formed around the outer side, and it went along and healed very nicely.

I have also used xeroform some on chafed lips in my own case, quickly healing them.

About two weeks ago a gentleman, seventy-nine years of age, called with a swollen face from a superior cuspid. I opened the tooth and the pus ran out freely. I syringed the canal thoroughly with trikresol and then pumped the xeroform into it, sealing it with wax over a rope of cotton. He returned at the end of ten days, and I filled the tooth permanently. I have done away with the use of iodoform, and I feel that xeroform is more reliable. I have been better satisfied with it than with anything else I have used to take the place of iodoform.

*Dr. J. G. Palmer.*—I would like to ask Dr. Watkins with regard to the case just before he went on his vacation, and also in the case of the gentleman seventy-nine years of age, why he should use both trikresol and xeroform if the latter has all the valuable features that are claimed for it. How does he know that xeroform did the work?

*Dr. Watkins.*—I used the trikresol as a carrying medium to make a base for the xeroform powder; trikresol is a good antiseptic, but, like other things of the kind, when it is shut up in a tooth for a time it loses its strength, and the tooth would need further treatment, whereas in connection with xeroform the one treatment is sufficient. I think it would make no difference how long xeroform was left in a canal; if left a year, I think the canal would be found to be perfectly aseptic at the end of that time.

*Dr. Benjamin Lord.*—I would like to ask Dr. Watkins what he would fill the canal with.

*Dr. Watkins.*—With gutta-percha.

*Dr. Lord.*—Dissolved gutta-percha?

*Dr. Watkins.*—No; little cones softened by heat and carried to the ends of the roots.

*The President.*—There are quite a number of substances similar to the one of which Dr. Watkins speaks. They belong to the group which comprises iodine, carbolic acid, creosote, and their derivatives. Carbolic acid, as is well known, is divisible into cresylic and phenic acids. The substance under discussion this evening is of the phenic acid group, and has been mostly used among the Germans. I have a pamphlet in my hand describing three of these compounds: betanaphtol-bismuth, phenol-bismuth, and tribromophenol-bismuth, all of which act, as is supposed, by splitting up into their component parts, the principal one of which is phenic acid, and this in turn does the work of antisepticizing the territory under treatment.

They are designed, as Dr. Watkins says, to take the place of iodoform, which, besides having a most offensive odor, is poisonous under certain conditions, and produces, when its systemic action is felt, loss of appetite, giddiness, and other unpleasant symptoms.

Iodoform is supposed to act by eliminating iodine, which is its active principle; but in xeroform the active principle is phenol.

Dr. Heuss explains that when xeroform is placed upon the skin with its acid reaction the drug is inert; that it is only when in contact with the living alkaline tissue fluids or with the mucous membrane that it becomes useful.

To obtain the fullest and quickest action from xeroform all sloughs, bits of coagulated blood, pus, and scales must be removed, and the powder applied directly to the surface of the wound; and it is only under these conditions that the division into parts takes place, the bismuth acting as a covering, while the phenol does its work, but so gradually that it is no longer toxic, as it would be if applied directly.

In France a substance called traumatol or iodocresine is used precisely as xeroform is recommended to be used.

Traumatol has as its basis cresylic acid in place of phenic, and it is thought by those who advocate its use that, as cresylic acid is in some respects probably the most powerful antiseptic known, it will prove to be the long-desired and much-hoped-for dressing for open wounds.

I have quite an account here of syphilitic cases treated with xeroform successfully, acting upon the principles given above.

I was hoping that Dr. Watkins would give us something more accurate in regard to the treatment of a closed root of a tooth, for, judging from the description which these little pamphlets give, we should have pretty nearly a stoppage of the pulp-canals in which xeroform might be placed.

How the action of xeroform can be differentiated from the action of the trikresol, with which it was mixed by Dr. Watkins, he fails to tell us.

If there is nothing further upon this point, we shall expect to see and listen to the demonstrations and explanations of Drs. Kimball and Lord in using soft gold.

*Dr. C. O. Kimball.*—I am afraid that I shall seem somewhat puerile in the elementary things which I have to bring before you, and yet after reading to one of our friends what I intended to say on the subject, and giving him a similar demonstration to that which I wish to give to you, I asked him if I were not too childish, and he said, "On the contrary, I wish for my sake that you would make it still more elementary, calling attention to the finer points of the work." That must be my excuse for offering a few words and a demonstration out of the mouth upon a method which some of us have practised for a great many years, and which is in its main features well known to all present.

Dr. Kimball then gave a demonstration of his method of preparing soft gold, and filled a cavity in a tooth out of the mouth, explaining meanwhile the details of the operation.

(For Dr. Kimball's paper, see page 211.)

*The President.*—Dr. Lord requested me to say that he regretted the necessity of going home at this hour, but that he would hold himself in readiness on a future occasion to speak on the subject.

Adjourned.

S. E. Davenport, D.D.S., M.D.S.,
*Editor The New York Institute of Stomatology.*

